# Energy expenditure associated with walking speed and angle of turn in children

**DOI:** 10.1007/s00421-018-3981-1

**Published:** 2018-09-05

**Authors:** Sam G. M. Crossley, Kelly A. Mackintosh, Rory P. Wilson, Leanne J. Lester, Iwan W. Griffiths, Melitta A. McNarry

**Affiliations:** 1 Applied Sport Technology Exercise and Medicine Research Centre, Swansea University, Swansea, Wales, UK; 2 Swansea Lab for Animal Movement, Biosciences, College of Science, Swansea University, Singleton Park, Swansea, Wales, UK; 3 School of Human Science, The University of Western Australia, Crawley, Australia

**Keywords:** Youth, Energy requirements, Velocity, Change of direction

## Abstract

**Purpose:**

Recent studies have suggested that turning is power intensive. Given the sporadic and irregular movement patterns of children, such findings have important implications for the assessment of true energy expenditure associated with habitual physical activity. The purpose of this study was to investigate the influence of walking speed and angle, and their interaction, on the energy expenditure of healthy children.

**Methods:**

20 children (10.1 ± 0.5 years; 10 boys) participated in the study. On two separate days, participants completed a turning protocol involving 3-min bouts of walking at one of the 16 speed (2.5, 3.5, 4.5, and 5.5 km h^− 1^) and angle (0°, 45°, 90°, and 180°) combinations, interspersed by 3 min seated rest. The movement involved 5 m straight walking interspaced with prescribed turns with speed dictated by a digital, auditory metronome. Breath-by-breath gas exchange was measured, in addition to tri-axial acceleration and magnetic field intensity recorded at 100 Hz.

**Results:**

Mixed models revealed a significant main effect for speed (*p* < 0.006) and angle (*p* < 0.006), with no significant interaction between speed and angle (*p* > 0.006). Significant differences to straight-line walking energy expenditure within speed were established for 3.5 and 5.5 km h^− 1^ for 180° turns (~ 13% and ~ 30% increase, respectively).

**Conclusion:**

These findings highlight the importance of accounting for the magnitude and frequency of turns completed when estimating children’s habitual physical activity and have significant implications for the assessment of daily energy expenditure.

## Introduction

Insufficient physical activity is one of the leading risk factors for global mortality, at least in part due to its association with obesity and non-communicable diseases (NCDs), such as cardiovascular disease, cancer, and diabetes (WHO 2017). However, despite the numerous physiological (Janssen and Leblanc 2010; Sothern et al. 1999) and psychosocial benefits (Eime et al. 2013; Nieman 2002) associated with physical activity, it is suggested that as little as 19% of boys and 16% of girls meet the current government guidelines of 60 min of moderate-to-vigorous physical activity every day (Townsend et al. 2015). A central tenet to these guidelines is the link between defined physical activity and energy expenditure.

The most accurate measure of an individual’s physical activity and energy expenditure is by assessing the body’s oxygen utilization and carbon dioxide production using indirect calorimetry methods (Levine 2005). However, this level of measurement is expensive and in-practical in free-living settings (Strath et al. 2013; Westerterp 2009), although does provide an essential criterion method by which to judge the accuracy and precision of smaller and less obtrusive devices such as accelerometers (Freedson et al. 2005; Rowlands 2007). Accelerometers have been widely utilized among researchers (Doherty et al. 2017; Lee and Shiroma 2014; Leung et al. 2017; Ward et al. 2005) as a result of their relatively accurate estimates of oxygen uptake ($\dot {V}{{\text{O}}_{\text{2}}}$; de Almeida Mendes et al. 2018; McGregor et al. 2009). Nevertheless, there are some limitations to accelerometer measurements as they are most commonly based upon linear regression models that emphasize that energy expenditure increases linearly with vertical accelerations (Freedson et al. 2012), which tend to discredit non-locomotive activities, such as turning (Bassett and John 2010; Chen et al. 2007; Van Remoortel et al. 2012). In this regard, children are problematic, since their movement is highly sporadic (Baquet et al. 2007; Sleap and Warburton 1996; Welk et al. 2000), which presents challenges to power-use determination protocols that typically require steady-state conditions (Reilly et al. 2004; Trost et al. 2011). Subsequently, the development of new multi-sensor devices that integrate both accelerometer and magnetometer measurements have been extensively used as a proxy of $\dot {V}{{\text{O}}_{\text{2}}}$ in humans (McNarry et al. 2017; Qasem et al. 2012; Weippert et al. 2013) as a result of the combined ability to capture additional information regarding how the body rotates during pathways that require turning (Williams et al. 2017) .

Turning is a fundamental movement within human locomotion and is particularly prevalent in children’s habitual physical activity patterns (Sleap and Warburton 1996). Even within adults, turning can make up 35–45% of all steps taken in a typical day (Glaister et al. 2007). While turning has not generally been considered to be associated with significant additional energetic costs over straight-line walking, this attitude is now changing (Dellal et al. 2010). According to Hamill et al. (1983), curved path locomotion or turning may subject individuals to unique stresses. For example, a study in 2011 suggested that 15% of the total energy expenditure during stair climbing can be attributed to turning in adults (Minetti et al. 2011), while Buchheit et al. (2011) reported marked physiological changes associated with turning; increased heart rate, blood lactate, and perceived exertion during intermittent shuttle run tests with a 180° turn compared to straight running. Similarly, it has been shown that completing 30 turns per minute at 3 km h^− 1^ elicits a similar energy expenditure to straight-line walking at 6 km h^− 1^ (Hatamoto et al. 2014). Furthermore, Wilson et al. (2013) extended these findings to consider a range of turning angles, demonstrating that as the angle of the turn increased, so did the associated energy expenditure. Specifically, a single 180**°** turn elicited the same energy expenditure as walking 5.88 m in a straight line at a velocity of 1.67 m s^− 1^ (6 km h^− 1^). To consider these findings as a function of walking speed, McNarry et al. (2017) reported a synergistic interaction between speed and angle in determining the energy expenditure associated with walking. A similar study investigated the energy expenditure of turning and walking in community-dwelling elderly, reporting that 180° turns were significantly more energy demanding than 90° turns (Justine et al. 2014). However, the applicability of these findings to children is questionable, not least due to their unique physiological and biomechanical structure (Andropoulos 2012). To date, no studies have specifically addressed the energy expenditure of turning in children. Such findings will have important implications for developing technology that enables a more precise unobtrusive assessment of children’s physical activity and intensity that is essential for detailed investigations of dose–response relationships between physical activity and health, the evaluation of interventions and enhancing an individual’s awareness of their physical state to enforce behaviour change.

The purpose of the present study was to investigate the influence of turn angle and walking speed on energy expenditure in children. We hypothesized that (1) as speed of walking increased, so too would the energy expenditure; (2) as angle of turn increased, so would the energy expenditure and that (3) walking speed and angle of turn would interact to modulate energy expenditure.

## Methods

### Participants and anthropometry

Twenty healthy children, aged 9–12 years (10.1 ± 0.5 years; 10 boys) were recruited from a local primary school. Child assent and parental or guardian consent were obtained prior to study participation. Body mass (Seca 876, Hamberg, Germany), stature (Holtain Stadiometer, Holtain Ltd), and sitting height (Holtain Sitting Height Stadiometer, Holtain Ltd) were measured to the nearest 0.1 kg and 0.1 cm, respectively. Sexual maturity was assessed by self-report using the indices of pubic hair described by Tanner (1962). Pre-pubertal status was defined as Tanner stage 1 (*n* = 12), with stage 2 being early pubertal (*n* = 4), Tanner stage 3 mid-pubertal (*n* = 4), Tanner stages 4 and 5 being late pubertal, and post pubertal, respectively (Chan et al. 2010). To provide an additional indicator of physical maturity, the age to peak height velocity equation devised by Mirwald et al. (2002) was used based on the measurement of standing and seated height, weight, and age to calculate maturity offset. Participants were asked to arrive at the laboratory in a rested state, at least 2-h postprandial. All procedures employed during this study were approved by Swansea University ethics committee and were conducted in accordance with the Declaration of Helsinki (ref: PG/2014/16).

### Incremental treadmill test

Participants were required to visit the laboratory on one occasion to perform an incremental treadmill test to volitional exhaustion for the determination of the gas exchange threshold (GET) and peak oxygen uptake ($\dot {V}{{\text{O}}_{{\text{2}}\,{\text{peak}}}}$). The children were first familiarised with walking and running on the treadmill at a range of speeds (4, 6, and 8 km h^− 1^) and with the testing equipment. To take into account the variation in biological ages, individual $\dot {V}{{\text{O}}_{{\text{2}}\,{\text{peak}}}}$ test speeds were calibrated by anchoring treadmill speeds to set Froude numbers (Houston et al. 2013). Specifically, treadmill speeds were calculated using the Froude number, gravity, and leg length equation (Minetti 2001). The initial stages were set at a 1% gradient (Jones and Doust 1996) and increased every 2 min, beginning with a walking speed equivalent to Froude 0.25. Subsequent increments were determined by the calculated difference between stage 1 and 2 speeds (~ 2 km h^− 1^) until maximal running velocity was achieved. At this point, the gradient was then increased by 1% every minute until volitional exhaustion was reached.

### Turning protocol

**Fig. 1:**
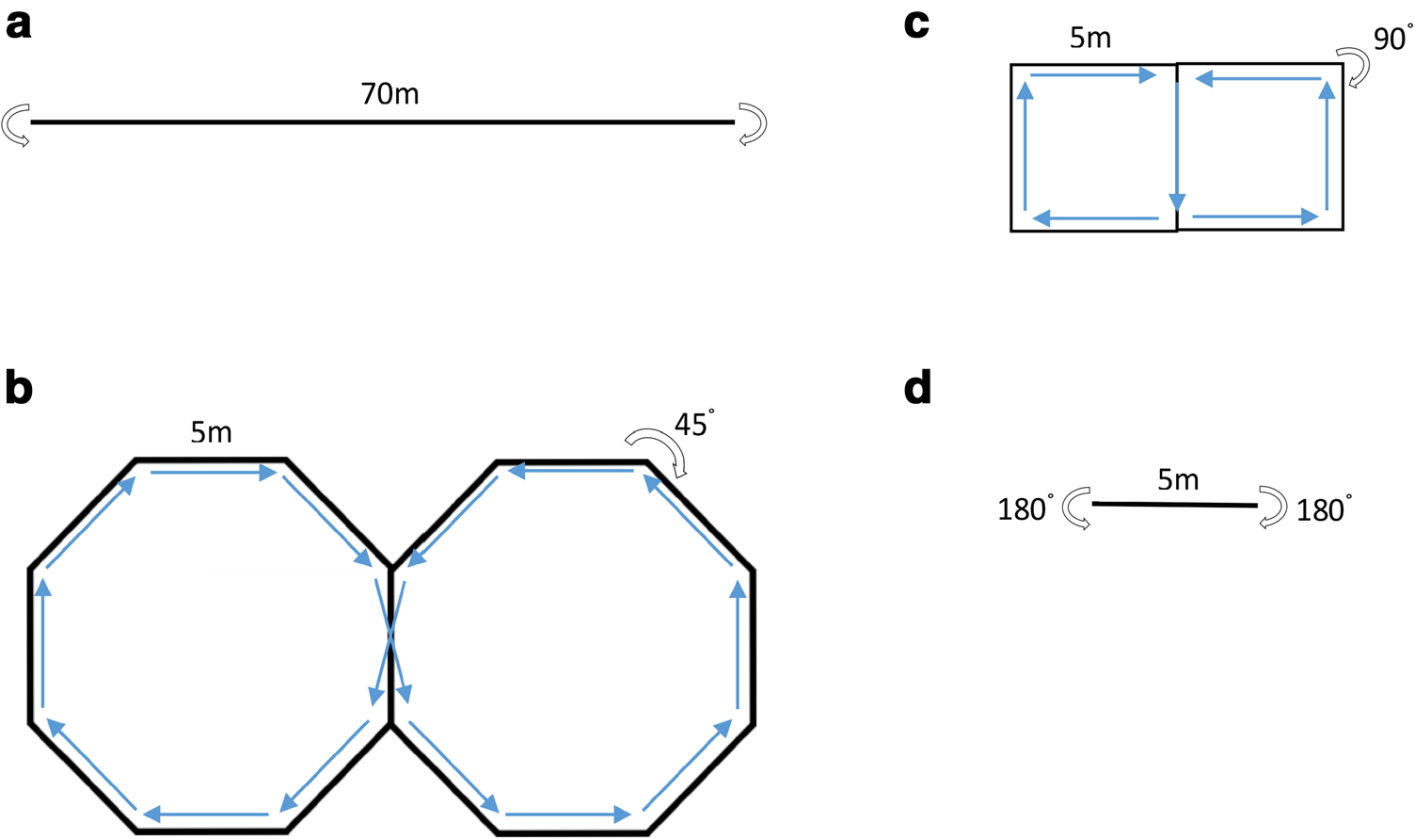
Experimental setup of the turning protocol showing 5 m straights interspersed by prescribed angle of turns **a** 0°, **b** 45°, **c** 90°, and **d** 180° with equal left and right hands turns

The second part of the testing involved completing a turning protocol, which was repeated on two occasions, separated by a minimum of 24 h. During this protocol, each participant was asked to complete 3-min bouts of walking interspersed by 3 min of seated rest. Each participant walked at four different walking speeds (2.5, 3.5, 4.5, and 5.5 km h^− 1^) in combination with four different turn angles (0°, 45°, 90°, and 180°), in a randomized order, as illustrated in Fig. 1. Specifically, each of the conditions involved 5 m straight walking stretches interspaced with prescribed turns with the speed dictated by a digital, auditory metronome. The auditory metronome sounded half-way along the 5 m straight and on the turns, so variability in speed within conditions was minimised. Furthermore, all participants were accompanied by one of the research team to act as a pacesetter. Each condition incorporated an equal number of left and right turns.

### Measurements

**Fig. 2:**
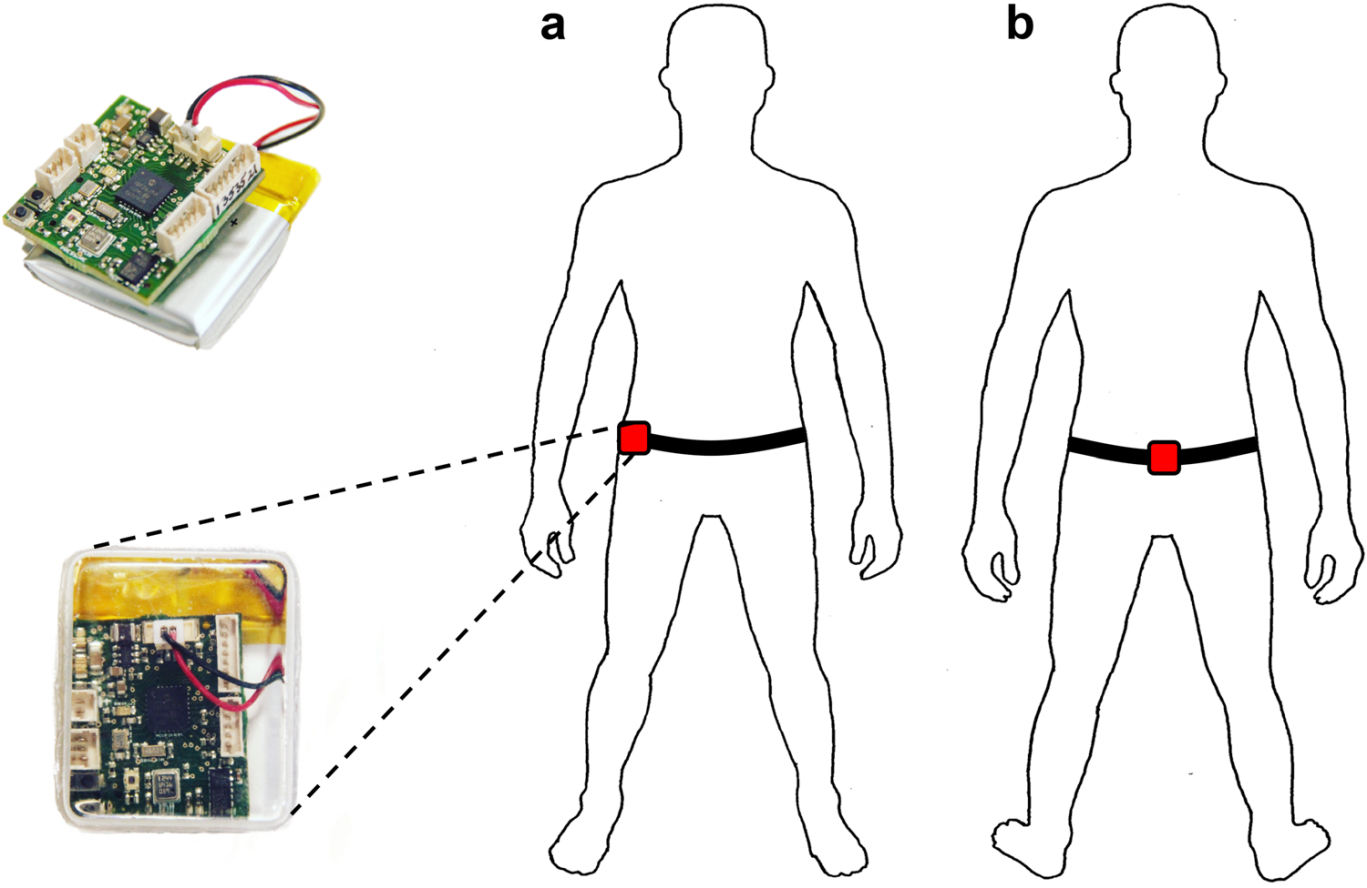
SLAM tracker device placement: **a** right mid-axilla line at the level of the iliac crest; **b** middle of the lower back

Throughout all the tests, gas exchange variables were measured on a breath-by-breath basis (MetaMax Cortex 3B, CORTEX Biophysik GmbH, Germany). Prior to each test, the gas analysers were calibrated using gases of known concentration and the turbine volume transducer was calibrated using a 3-L syringe (Hans Rudolph, Kansas City, MO, USA). The delay in the capillary gas transit and analyser rise time was accounted for relative to the volume signal, thereby time-aligning the concentration and volume signals. In addition, two custom-built tri-axial accelerometers and magnetometers (SLAM Tracker, Wildbyte Technologies Ltd, Swansea, UK), measuring at 100 Hz on all channels, were worn by participants: one tag was worn on the right mid-axilla line at the level of the iliac crest and one tag at the middle of the lower back (see Fig. 2), in accord with the previous methods (McNarry et al. 2017).

### Data analysis

$\dot {V}{{\text{O}}_{{\text{2}}\,{\text{peak}}}}$ was defined as the highest 10-s stationary average during the incremental exercise test. The GET was determined by the *V*-slope method (Beaver et al. 1986) as the point at which carbon dioxide production began to increase disproportionately to $\dot {V}{{\text{O}}_{\text{2}}}$, as identified using the purpose-written software developed using LabVIEW (National Instruments, Newbury, UK). Mean absolute $\dot {V}{{\text{O}}_{\text{2}}}$ values for defining steady state for each individual speed and turn were taken from the last 45 s of each 3-min turning condition (Wilson et al. 2013). Analyses of turning energy expenditure were based on the premise that the additional turn cost was superimposed on the baseline of straight-line travel. Specifically, the difference in $\dot {V}{{\text{O}}_{\text{2}}}$ between straight-line walking (0°) at each velocity relative to the $\dot {V}{{\text{O}}_{\text{2}}}$ associated with walking at 45°, 90°, or 180° turns was defined as the additional cost of turning. The net energy cost of walking (*C*_r_) was calculated from participants’ absolute $\dot {V}{{\text{O}}_{\text{2}}}$ values normalized per unit body mass for each experimental condition and divided by the walking speed converted to meters per minute (m/min), expressed as ml O_2_·kg^− 1^ km^− 1^. To account for body size, the procedures proposed by Welsman and Armstrong (2000) were used to calculate the allometric scaling coefficient for absolute $\dot {V}{{\text{O}}_{\text{2}}}$ for turning, straights, and $\dot {V}{{\text{O}}_{{\text{2}}\,{\text{peak}}}}$. First, the data were logarithmically transformed to determine the allometric relationship between body mass, $\dot {V}{{\text{O}}_{\text{2}}}$ and $\dot {V}{{\text{O}}_{{\text{2}}\,{\text{peak}}}}$. Common allometric exponents were confirmed, which were then linearly regressed to obtain a coefficient and then expressed using the formula:

$${\text{Scaled}}\,\dot {V}{{\text{O}}_{\text{2}}}=Y/{X^b},$$

where *Y* is the participants $\dot {V}{{\text{O}}_{\text{2}}}$ for a turn, straight or $\dot {V}{{\text{O}}_{{\text{2}}\,{\text{peak}}}}$, *X* is the body mass of the participant, and *b* is the scaling coefficient derived from the linear regression.

Significant absolute and scaled $\dot {V}{{\text{O}}_{\text{2}}}$ outliers were established using box plots with Tukeys 1.5 multiplier of the standard deviation (Tukey 1977). Subsequently, one participant was excluded from further analyses due to significant outliers identified in test 1.

The raw accelerometer data were first converted to dynamic body acceleration (DBA) by smoothing each channel to derive the static acceleration using a running mean over 2 s (Shepard et al. 2008). The static acceleration was then subtracted from the raw acceleration data (Gleiss et al. 2011), resulting in values for dynamic acceleration that were all converted to positive values. These values for DBA were summed vectorially to provide ‘vectorial dynamic body acceleration’ (VeDBA):

$${\text{VeDBA}}=\sqrt {(A_{x}^{2}+A_{y}^{2}+A_{z}^{2})} ,$$

where *A*_*x*_, *A*_*y*_, and *A*_*z*_ are the derived dynamic accelerations at any point in time corresponding to the three orthogonal axes of the accelerometer (Qasem et al. 2012). VeDBA has been used extensively as a proxy for $\dot {V}{{\text{O}}_{\text{2}}}$ in a suite of vertebrates (cf. Halsey et al. 2011), including humans (McNarry et al. 2017; Qasem et al. 2012; Weippert et al. 2013), with appreciable success. However, many aspects of the particulars of the acceleration data recorded in such trials (e.g., lateral versus forward–backward) as well as the effect of incline (cf. Bidder et al. 2012) and tag mounting have not been examined critically, so our use of this metric has to be seen within this context.

Using the middle minute and overall 3-min bout, both mean and summed VeDBA were derived for each individual turn and straight for each condition. The individual turns and straight sections were analyzed using a custom developed C++ software (DDMT Wildbyte Technologies Ltd, Swansea, UK) specially designed for visualizing the accelerometry and magnetometry traces to identify inter alia turns via systematic changes in the magnetometry data (Williams et al. 2017).

### Statistics

A Sharpiro–Wilks test was used to confirm data normality. Repeated measures linear mixed-effects models with a Tukey’s test of post hoc means test were conducted using IBM SPSS Statistics 22 (Chicago, IL, USA) to account for the repeated measures and correlated nature of the data to determine the influence of, and interaction between, independent variables walking speed and angle, with the dependent variables expressed as *C*_r_, absolute $\dot {V}{{\text{O}}_{\text{2}}}$, scaled $\dot {V}{{\text{O}}_{\text{2}}}$, and mean VeDBA (Halsey et al. 2009; Qasem et al. 2012). All condition combinations for turning $\dot {V}{{\text{O}}_{\text{2}}}$ (absolute or scaled) and mean VeDBA (straight or turn) were placed into one mixed model analysis with covariates (sex, stature, and cardiorespiratory fitness) to determine their modulatory effect. The Tukey–Ciminera–Heyse (TCH; Sankoh et al. 1997; Tukey et al. 1985) procedure was used to determine an adjusted significance value for all comparisons within the mixed model to account for experimentwise error. A Pearson product–moment correlation coefficient was used to determine any associations between the dependent variables $\dot {V}{{\text{O}}_{\text{2}}}$ and straight and turn VeDBA, including all covariates. All data are presented as mean ± SD.

## Results

The characteristics of the sample population are displayed in Table 1. Boys were significantly heavier, taller and demonstrated a higher peak $\dot {V}{{\text{O}}_{\text{2}}}$ than girls, in both absolute and scaled terms. All participants were found to be either pre-pubertal or early-to-mid-pubertal according to the self-reported Tanner stages. To determine the relationship between walking speed and angle on energy expenditure, a mixed model was used to examine the main effects of speed and angle, and the interaction of speed and angle while controlling for sex. Our post hoc evaluations indicated that the empirical technique utilized fully met the intended objectives. The TCH procedure resulted in an adjusted significance value of 0.006(6), calculated from 60 pairwise comparisons.

**Table 1:** Participant characteristics

	Total	Boys	Girls
*n*	19	9	10
Age, years	10.1 ± 0.5	10.2 ± 0.6	10.0 ± 0.3
Tanner stages, %	60% T1, 20% T2, 20% T3	30% T1, 40% T2, 30% T3	90% T1, 10% T3
Years to PHV, years	− 2.4 ± 0.8	− 3.03 ± 0.3	− 1.9 ± 0.6
Stature, m	1.39 ± 0.07	1.42 ± 0.05	1.38 ± 0.09^a^
Body mass, kg	33.7 ± 5.7	35.03 ± 3.8	32.6 ± 7.0^a^
BMI, kg m^− 2^	17.1 ± 2.3	17.3 ± 1.7	17.2 ± 2.8
Peak $\dot {V}{{\text{O}}_{\text{2}}}$, l min^− 1^	1.63 ± 0.39	1.79 ± 0.44	1.49 ± 0.29^a^
Scaled peak $\dot {V}{{\text{O}}_{\text{2}}}$, l kg^− 0.79^ min^− 1^	100.8 ± 18.8	98.8 ± 17.4	102.5 ± 20.7^a^
GET, l min^− 1^	1.03 ± 0.25	1.17 ± 0.25	0.90 ± 0.18

Mean ± SD

*PHV* peak height velocity, *T1* Tanner stage 1, *T2* Tanner stage 2, *T3* Tanner stage 3, *BMI* body mass index, $\dot {V}{{\text{O}}_{\text{2}}}$ oxygen uptake, *GET* gas exchange threshold

^a^Significant difference between boys and girls (*p* < 0.05)

The values of *C*_r_, for all participants under all experimental conditions, are reported in Table 2. The *C*_r_ straight (0°) decreases with speed to attain a significant (*p* < 0.006) minimum energy expenditure at 5.5 km h^− 1^ compared to 2.5 km h^− 1^. The effect of 45° and 90° turns on *C*_r_ is relatively minor. However, 180° turns at speeds 3.5, and 5.5 km h^− 1^, significantly greater *C*_r_ (*p* < 0.006) were established when compared to straight-line walking within speeds. More specifically, for a 180° angle, speed increased *C*_r_ by ~ 7% at 2.5 km h^− 1^, to ~ 13% at 3.5 km h^− 1^ (*p* < 0.006), ~ 14% at 4.5 km h^− 1^, to attain ~ 30% increase at 5.5 km h^− 1^ (*p* < 0.006).

**Table 2:** Values for net energy cost of walking and turning

Speed (km h^− 1^ = m min^− 1^)	$\dot {V}{{\text{O}}_{\text{2}}}$ (*C*_r_, ml O_2_ kg^− 1^ km^− 1^)			
	0°	45°	90°	180°
2.5 = 41.67	0.28 ± 0.05	0.29 ± 0.05	0.30 ± 0.05	0.30 ± 0.06
3.5 = 58.33	0.23 ± 0.04^a^	0.23 ± 0.04^a^	0.24 ± 0.04^a^	0.26 ± 0.05^b^
4.5 = 75.00	0.21 ± 0.04^a^	0.21 ± 0.04^a^	0.21 ± 0.04^a^	0.24 ± 0.04^a^
5.5 = 91.67	0.20 ± 0.04^a^	0.20 ± 0.03^a^	0.20 ± 0.04^a^	0.26 ± 0.04^b^

Mean ± SD

$$\dot {V}{O_2}$$ oxygen uptake

^a^Significant difference to 2.5 km h^− 1^ within angle (*p* < 0.006)

^b^Significant difference to straight-line walking within speed (*p* < 0.006)

**Fig. 3:**
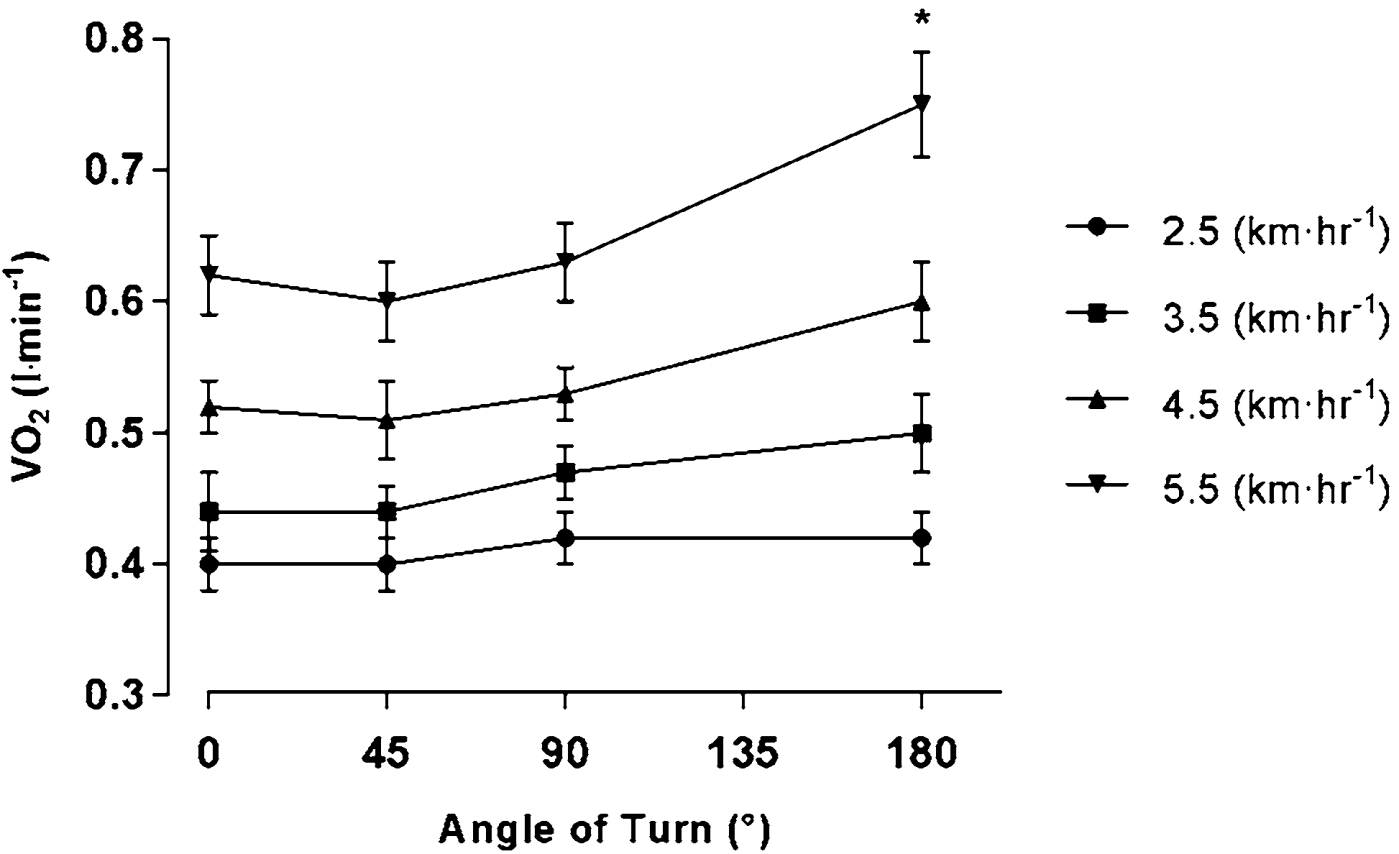
Interaction between speed and angle on absolute $\dot {V}{{\text{O}}_{\text{2}}}$, displaying SEM. ‘Asterisk’ indicates a significant difference in energy expenditure of turning relative to straight-line walking at 5.5 km h^− 1^ (*p* < 0.006)

Participants’ mean absolute and scaled $\dot {V}{{\text{O}}_{\text{2}}}$ are reported in Table 3. There was a significant main effect for speed (*F* = 101.13, *p* < 0.006) and turn angle (*F* = 11.52, *p* < 0.006) on absolute $\dot {V}{{\text{O}}_{\text{2}}}$, with no significant interaction between speed and angle (*F* = 2.01, *p* > 0.006; Table 5, see “Appendix”), with similar effects still observed when scaled to account for body size (speed, *F* = 106.30, *p* < 0.006; angle, *F* = 13.96, *p* < 0.006; speed and angle, *F* = 168.14, *p* > 0.006). As shown in Fig. 3, increasing speed was found to increase $\dot {V}{{\text{O}}_{\text{2}}}$ within a turn angle, but significant increases in $\dot {V}{{\text{O}}_{\text{2}}}$ due to turning relative to straight-line walking were only observed at 180° at the highest speed. There were no significant differences between boys and girls $\dot {V}{{\text{O}}_{\text{2}}}$ across all conditions (*F* = 6.26, *p* > 0.006), regardless of the method of expression for $\dot {V}{{\text{O}}_{\text{2}}}$. Stature (*F* = 26.27, *p* < 0.006) and $\dot {V}{{\text{O}}_{{\text{2}}\,{\text{peak}}}}$ (*F* = 24.53, *p* < 0.006) were significant predictors of $\dot {V}{{\text{O}}_{\text{2}}}$ during each condition for both sexes, although when condition $\dot {V}{{\text{O}}_{\text{2}}}$ was scaled, scaled $\dot {V}{{\text{O}}_{{\text{2}}\,{\text{peak}}}}$ was no longer a significant predictor (*F* = 0.98, *p* > 0.006). The significant predictors for absolute and scaled $\dot {V}{{\text{O}}_{\text{2}}}$ models on speed, angle, and their interaction are shown in Table 5 (see "Appendix").

**Table 3:** Mean energy expenditure during each combination of walking velocity and angle

	0°	45°	90°	180°
Absolute $\dot {V}{{\text{O}}_{\text{2}}}$ (l min^− 1^)				
2.5 km h^− 1^	0.40 ± 0.10	0.40 ± 0.10	0.42 ± 0.10	0.42 ± 0.10
3.5 km h^− 1^	0.44 ± 0.11^a^	0.44 ± 0.10	0.47 ± 0.11	0.50 ± 0.13^a,b^
4.5 km h^− 1^	0.52 ± 0.11^a^	0.51 ± 0.12^a^	0.53 ± 0.11^a^	0.60 ± 0.15^a,b^
5.5 km h^− 1^	0.62 ± 0.13^a^	0.60 ± 0.13^a^	0.63 ± 0.15^a^	0.75 ± 0.17^a,b^
Scaled $\dot {V}{{\text{O}}_{\text{2}}}$ (l kg^−0.79^ min^−1^)				
2.5 km h^− 1^	24.75 ± 4.40	25.08 ± 4.40	26.25 ± 4.34	26.00 ± 4.99
3.5 km h^− 1^	27.62 ± 5.18^a^	27.56 ± 5.14	29.27 ± 4.58^a^	31.26 ± 6.06^a^
4.5 km h^− 1^	32.84 ± 5.55^a^	32.06 ± 6.10^a^	33.41 ± 5.73^a^	37.39 ± 6.71^a^
5.5 km h^− 1^	38.73 ± 7.06^a^	37.79 ± 6.51^a^	39.41 ± 7.26^a^	47.11 ± 8.41^a,b^

Mean ± SD

$$\dot {V}{O_2}$$ oxygen uptake

^a^Significant difference to 2.5 km h^− 1^ within angle (*p* < 0.006)

^b^Significant difference to straight walking within speed (*p* < 0.006)

A significant main effect was found for speed (*F* = 548.49, *p* < 0.006) but not for angle (*F* = 3.66, *p* > 0.006) on straight mean VeDBA, with significant effects found for both speed and angle on turn mean VeDBA (speed: *F* = 724.88, *p* < 0.006; angle: *F* = 4.96, *p* < 0.006). Speed and angle had no significant interaction effect on mean VeDBA during either straight (*F* = 0.82, *p* > 0.006) or turns (*F* = 0.99, *p* > 0.006). Specifically, both straight and turning mean VeDBA increased with speed (Table 4), but no significant effects were found (Table 6, see "Appendix"). For straight walking VeDBA, there were no significant predictors (sex: *F* = 0.40, *p* > 0.006; $\dot {V}{{\text{O}}_{{\text{2}}\,{\text{peak}}}}$ *F* = 2.62, *p* > 0.006; scaled $\dot {V}{{\text{O}}_{{\text{2}}\,{\text{peak}}}}$ *F* = 1.53, *p* > 0.006), with turning VeDBA significantly predicted by sex (*F* = 14.20, *p* < 0.006) with both $\dot {V}{{\text{O}}_{{\text{2}}\,{\text{peak}}}}$ (*F* = 6.25, *p* > 0.006) and scaled $\dot {V}{{\text{O}}_{{\text{2}}\,{\text{peak}}}}$ (*F* = 2.67, *p* > 0.006) not significantly predicting (Table 6, see "Appendix").

**Table 4:** Mean VeDBA, straight, and turn mean VeDBA during each combination of walking velocity and angle

	0°	45°	90°	180°
Mean VeDBA (*g*)				
2.5 km h^− 1^	0.22 ± 0.02	0.21 ± 0.02	0.22 ± 0.03	0.22 ± 0.03
3.5 km h^− 1^	0.28 ± 0.04^a^	0.29 ± 0.05^a^	0.29 ± 0.04^a^	0.31 ± 0.04^a,b^
4.5 km h^− 1^	0.41 ± 0.07^a^	0.40 ± 0.07^a^	0.41 ± 0.07^a^	0.44 ± 0.06^a^
5.5 km h^− 1^	0.60 ± 0.10^a^	0.59 ± 0.11^a^	0.59 ± 0.13^a^	0.63 ± 0.11^a^
Straight mean VeDBA (*g*)				
2.5 km h^− 1^	0.21 ± 0.02	0.22 ± 0.03	0.22 ± 0.03	0.22 ± 0.03
3.5 km h^− 1^	0.29 ± 0.05^a^	0.29 ± 0.04^a^	0.31 ± 0.04^a^	0.32 ± 0.05^a,b^
4.5 km h^− 1^	0.40 ± 0.07^a^	0.41 ± 0.07^a^	0.44 ± 0.06^a^	0.44 ± 0.07^a^
5.5 km h^− 1^	0.59 ± 0.11^a^	0.59 ± 0.13^a^	0.63 ± 0.11^a^	0.63 ± 0.11^a^
Turn mean VeDBA (*g*)				
2.5 km h^− 1^	0.22 ± 0.06^a^	0.22 ± 0.03	0.22 ± 0.03	0.22 ± 0.03
3.5 km h^− 1^	0.30 ± 0.07^a^	0.30 ± 0.05^a^	0.29 ± 0.05^a^	0.31 ± 0.06^a,b^
4.5 km h^− 1^	0.39 ± 0.06^a^	0.41 ± 0.08^a^	0.40 ± 0.08^a^	0.44 ± 0.09^a^
5.5 km h^− 1^	0.56 ± 0.11^a^	0.62 ± 0.13^a^	0.59 ± 0.13^a^	0.63 ± 0.12^a^

Mean ± SD

*VeDBA* vectorial dynamic body acceleration

^a^Significant difference to 2.5 km h^− 1^ within angle (*p* < 0.006)

^b^Significant difference to straight walking within speed (*p* < 0.006)

### Pearson product–moment correlation coefficient

There were significant correlations between $\dot {V}{{\text{O}}_{\text{2}}}$ and straight (*r*^2^ = 0.51; *p* < 0.006) and turning VeDBA (*r*^2^ = 0.54; *p* < 0.006), with a weaker but statistically significant relationship between $\dot {V}{{\text{O}}_{{\text{2}}\,{\text{peak}}}}$ (*r*^2^ = 0.30; *p* < 0.006) and stature (*r*^2^ = 0.32; *p* < 0.006).

## Discussion

This is the first study to consider the energy expenditure of turning in children, demonstrating that as speed increases for any given angle of turn, the associated energy expenditure also increases. However, the extent to which angle contributed to an increased energetic demand was dependent upon the degree of the angle and walking speed. Specifically, increasing angles of turn and increasing walking speeds raised the energy expenditure, with 180° turns requiring a significantly greater energy expenditure than 45° or 90° turns. The findings presented highlight the importance of accounting for the magnitude of turn angle and the frequency of turns completed when estimating the habitual physical activity and energy expenditure of children; a failure to do so is likely to lead to erroneous conclusions regarding daily energy expenditure estimated from accelerometry data.

Numerous studies have highlighted the importance of accounting for turning, as well as the physiological and the biomechanical effects of turning when compared to straight-line locomotion in adults (Akram et al. 2010; Buchheit et al. 2012; Dellal et al. 2010; Hatamoto et al. 2013, 2014; Huxham et al. 2006; Justine et al. 2014; Orendurff et al. 2006; Patla et al. 1999; Wilson et al. 2013). A recent study found that when shuttle run distance was reduced from 7.0 to 3.5 m and completed at the same average running speed, the 3.5 m shuttles induced a greater physiological response (Bekraoui et al. 2012). Hatamoto et al. (2014) found that this greater physiological demand occurred even during walking velocities as low as 3 km h^− 1^. The present study extends these findings to children, demonstrating that *C*_r_ in a straight line decreased with speed to a minimum energy expenditure attained at 5.5 km h^− 1^ (1.5 m s^−1^). These findings align with the previous research, highlighting that human walking displays a U-shaped relationship between walking speed and energy expenditure of transport (Sparrow 2000; Willis et al. 2005). The optimal speed of walking is frequently cited to be between 4.8 (1.3 m s^−1^) and 6 km h^− 1^ (1.7 m s^−1^), in which the energy expenditure per unit distance travelled is minimised (Bastien et al. 2005; Zarrugh et al. 1974). Furthermore, the present findings regarding C_r_ to straight-line walking can be closely matched to Waters and Mulroy‘s (1999) regression equation for children’s $\dot {V}{{\text{O}}_{\text{2}}}$ by dividing the walking speed by m/min (following: $\dot {V}{{\text{O}}_{\text{2}}}$ cost = 0.188 + 2.61/*S*).

More importantly, the current findings showed that 180° turns had a significantly greater energy expenditure per unit body mass and distance within speeds 3.5 km h^− 1^ and 5.5 km h^− 1^ exhibiting a 13% and 30% increase in energy expenditure, respectively, when compared to straight-line walking at the same speed. The resultant increase in energy expenditure of a 180° turn could be partly explained by the greater braking (deceleration) and propulsive (acceleration) forces encountered during turning (Schot et al. 1995). Specifically, Schot et al. (1995) found that a 90° turn experiences a greater acceleration phase, because an individual has to begin moving from a near stand-still position, whereas a 45° turn encompasses some of the residual incoming motion prior to the turn. According to Havens and Sigward (2015), larger turn angles lead to greater alterations for both deceleration and translation subtasks. Therefore, it could be postulated that a greater angle, such as a 180° turn, would experience larger deceleration and accelerations (Havens and Sigward 2015). As such, these findings may, in part, explain why paediatric populations with neuromuscular pathologies have trouble in turning, given their postural stability problems (Kenis-Coskun et al. 2016). In contrast, a recent study investigating the metabolic power of turning in youth soccer players concluded that turning (45° and 90°), while running is less metabolically demanding than straight-line running (Hader et al. 2016). It was concluded that this lower metabolic demand of turning may have been directly related to the very low energy demands of the deceleration phase during the turn that may not be compensated by the increased requirement for the re-acceleration phase. However, the study was limited using an indirect approach to estimate the energy demands of turning, which may ignore other non-locomotor muscles involved with turning (e.g., upper body and back muscles; Buchheit et al. 2010a, b). More research is warranted to investigate and strengthen our understanding of the energy expenditure associated with turning in child populations.

It is important to acknowledge that children are not “mini-adults” (Armstrong and Welsman 1997), mostly due to their anatomical and physiological differences when compared to adults (Andropoulos 2012). Given these biomechanical and physiological differences are likely to influence the energy expenditure of turning in children, the applicability of the previous findings in adults to children must be questioned, even though the physics of force generation needed for turns makes increased energy expenditure in a turn inevitable. More specifically, however, covariates such as age, stature, training status, and turning technique (Buchheit et al. 2011; Zadro et al. 2011) may have a significant role in determining the energy expenditure associated with any given task and may lead to discrepancies when comparing adults to children. McNarry et al. (2017) reported a synergistic interaction between speed and angle, with 90° and 180° turns associated with a significantly greater energy cost at walking speeds of 4.5 and 5.5 km h^−1^. In comparison, the present study revealed that there was no significant interaction between speed and angle, although there was a trend similar to that of the adults, with a walking speed of 5.5 km h^−1^ at 180° turn associated with the greatest energy expenditure when compared to all other combinations. In more detail, the mean $\dot {V}{{\text{O}}_{\text{2}}}$ for an adult turning 180° at a speed of 5.5 km h^−1^ was 1.54 ± 0.36 l min^−1^ (McNarry et al. 2017), which is substantially more than the value observed in the present study of 0.75 ± 0.17 l min^−1^. This discrepancy may be explained by the much larger stature of adults when compared to young children. Adults larger skeletal structure and muscle size is likely to change the moment arm length, both of which will result in increased energy costs (O’Brien et al. 2009). As Buchheit et al. (2011) found that shorter team sport players demonstrated less effect of a 180° change of direction than their taller counterparts. Lower angles of turn are associated with greater balance and stability as a proper support base is established, therefore, requiring less energy to turn (Justine et al. 2014). This is demonstrated in the present study, whereby children’s stature was a significant predictor for estimating the energy expenditure of turning, as supported by a similar relationship within adults (McNarry et al. 2017). It could, therefore, be postulated that children’s shorter moment arms and lower centre of gravity would lead to a reduced energy expenditure of turning when compared to taller adults.

The ability to maintain balance is essential for carrying out activities such as walking and a crucial component while turning. Dynamic postural control (i.e., balance) is defined as the ability to keep the centre of gravity within the base of support while performing a task in a stable condition (Winter et al. 1990). The previous studies observe deficits in postural control in children when compared to young healthy adults (Bosco and Komi 1980; Hytönen et al. 1993; Schärli et al. 2013), potentially due to a smaller base of support, which would be anticipated to be associated with a greater energy expenditure during turning. Furthermore, Geldhof et al. (2006) found better postural control in girls compared to boys between the ages of 9–10 years, which could explain the sex differences observed in the present study. As described by Hase and Stein (1999), there are two types of turn embedded into locomotion, one being the step turn and the other the spin turn. Step turns are biomechanically more efficient (Patla et al. 1991; Taylor et al. 2005) and offer greater stability (Taylor et al. 2005) than spin turns. When observing turning strategies in adult populations, step turns are most commonly reported in both laboratory (Patla et al. 1991) and non-laboratory environments (i.e., home or community; Glaister et al. 2007). Although findings from Dixon et al. (2013) is limited to laboratory settings, evidence suggests that children tend to adopt spin turns, with this adoption likely to be dependent on increasing gait velocity. Research suggests that spin turns limit the size of the moving base of support (Akram et al. 2010), which consequently leads to reduced stability, increasing the physiological strain on both lower limb (Hader et al. 2016) and upper body muscles (Buchheit et al. 2010). Therefore, it could be postulated that the increasing demands of a turn, such as a 180° at a speed of 5.5 km h^−1^, may expose children’s gait immaturity and concurrently lead to the adoption of the more complex turning sub-strategies identified within adult populations (Dixon et al. 2013). Although turning strategies and dynamic postural control were not accounted for in the present study, it is important to consider that turning strategies may have varied between children, affecting balance and consequently the variance of the values observed for energy expenditure. Therefore, further work on the energy expenditure of spin and step turn strategies is warranted in non-laboratory-based environments.

Some of the present study’s findings have implications for assessing children’s movement patterns in both habitual and health contexts. As highlighted by McNarry et al. (2017), it is important to account for the number of turns during a clinical six-minute walking test (6MWT), designed to measure both adults’ (Veloso-Guedes et al. 2011) and children’s (Geiger et al. 2007) functional exercise capacity. McNarry et al. (2017) highlight that the 6MWT varies due to limited space and resources, which consequently results in distances ranging from 20 to 50 m being used (Lipkin et al. 1986; Troosters et al. 1999), subsequently altering the frequency of turns completed from as much as 12 to 32 turns (Chetta et al. 2006). These methodological differences are likely to affect the reliability of aerobic capacity assessment using this method, especially for paediatric populations which have a restricted gait ability, such as those with cerebral palsy who show greater physiological cost of walking compared to healthy children (Liao et al. 1997). Nevertheless, a more recent study suggested that slow jogging with turns could be an effective exercise prescription to promote physical activity and fitness in inactive-healthy individuals and those who are overweight or obese. Specifically, Araki et al. (2017) demonstrated that walking at 4.2 km h^−1^, that is equal to 3 METs (light-intensity), to jogging at the same speed with turns, increased the intensity to 8 METs (vigorous intensity), which resulted in a 2.7-fold increase in energy expenditure. Moreover, Araki et al. (2017a, b) showed that slow walking (2.7 km h^−1^) became moderately intense (4 METs) when turns were incorporated. Therefore, including turns may be an effective method by which to increase the amount of physical activity that inactive individuals perform, to lose weight and increase fitness. However, there is a paucity of evidence to support the use of turning as a health promotion intervention, especially in children, therefore, warranting further investigation.

The findings in the present study question the previously assessed and validated accuracy of pedometers, gyroscopes, and accelerometers (Crouter et al. 2003; Esliger et al. 2007; Le Masurier and Tudor-Locke 2003; Mansfield and Lyons 2003; Rueterbories et al. 2010; Salarian et al. 2004), as the majority of the protocols performed during these studies have been relatively simple, with long periods of linear treadmill-based locomotion that are not true to the nature of habitual physical activity patterns. Indeed, these treadmill-based prediction equations may be a contributing factor to the poor accuracy of energy expenditure calculations during daily activities (Eisenmann et al. 2004; Fortune et al. 2014). In this respect, the magnetometer utilized in the present study provides additional information on how the body rotates (Williams et al. 2017), so when in conjunction with accelerometers, it will provide more context. Indeed, using the current methods to establish VeDBA from analysing the accelerometer and magnetometer traces proved to be relatively accurate in predicting energy expenditure expressed as $\dot {V}{{\text{O}}_{\text{2}}}$, aligning with the previous research (McNarry et al. 2017; Qasem et al. 2012; Weippert et al. 2013). However, from the present findings, it is apparent that there is a dissociation between VeDBA and turn angle, whereby increasing the angle of turn was not associated with significant increase in VeDBA. Similar findings were reported in adults by McNarry et al. (2017) arguing that this dissociation of VeDBA when turning could be a result of the complex and individual specific interaction between the surge, heave, and sway components of DBA as well as the muscular effort involved in generating forces without the dynamism of straight locomotion. Although little is known about the benefits of including such magnetometry-derived data on the accuracy of energy expenditure prediction, the present study highlights this collective measurement as an area that warrants further investigation, especially in children who are characterised by spontaneous and transitory movement patterns (Stone et al. 2009), such as football (Fjørtoft et al. 2009) and chasing games (Sleap and Warburton 1996) that likely involve a considerable number of turns.

It is important to note certain limitations of the current study. As this was the first study to investigate the energy expenditure of turning in children, findings should be taken with caution, due to the limited sample size and lack of evidence on the influence of children’s growth and maturation on the energy demands of turning, warranting further investigation. In addition, the highly controlled nature of this study may limit the generalisability of the findings and its ecological validity.

## Conclusions

In the present study, we found that the energy expenditure of turning, while walking was significantly greater at speeds of 3.5, 4.5 and 5.5 km h^−1^ at a turn angle of 180° in children. The study demonstrated the impact of turn angle and walking speeds on the energy expenditure of turning, with stature adding additional determinants of the demand. More research is warranted on running speeds and the effect of turning technique on the energy expenditure of turning. These findings highlight the importance of accounting for the costs of turning in children, with implications for both sporting, habitual physical activity, and health-related contexts, where turning is a fundamental part of movement.

## Appendix

See Tables 5 and 6.

**Table 5:** Absolute and scaled $\dot {V}{{\text{O}}_{\text{2}}}$ models

	Absolute $\dot {V}{{\text{O}}_{\text{2}}}$ (l min^− 1^)			Scaled $\dot {V}{{\text{O}}_{\text{2}}}$ (l kg^− 0.79^ min^− 1^)		
	Estimate (*β*)	SE	Sig.	Estimate (*β*)	SE	Sig.
Parameter	Speed					
5.5 km h^−1^	0.23	0.03	0.007	13.87	1.95	0.00^a^
4.5 km h^−1^	0.14	0.02	0.00^a^	8.78	1.48	0.00^a^
3.5 km h^−1^	0.07	0.02	0.002^a^	4.06	1.19	0.00^a^
2.5 km h^−1^	1.00	–	–	1.00	–	–
Parameter	Angle					
180°	0.02	0.02	0.38	1.26	1.39	0.37
90°	0.03	0.02	0.10	2.68	1.17	0.03
45°	0.10	0.02	0.62	0.89	1.20	0.46
0°	1.00	–	–	1.00	–	–
Parameter	Speed × angle					
Speed 5.5 × angle 180°	0.14	0.05	0.005^a^	8.84	2.89	0.003^a^
Speed 5.5 × angle 90°	− 0.32	0.04	0.48	− 1.95	2.65	0.46
Speed 5.5 × angle 45°	− 0.02	0.04	0.67	− 1.01	2.60	0.70
Speed 5.5 × angle 0°	1.00	–	–	1.00	–	–
Speed 4.5 × angle 180°	0.05	0.04	0.17	3.59	2.27	0.12
Speed 4.5 × angle 90°	− 0.03	0.03	0.40	− 2.11	2.06	0.31
Speed 4.5 × angle 45°	− 0.02	0.03	0.50	− 1.33	2.07	0.52
Speed 4.5 × angle 0°	1.00	–	–	1.00	–	–
Speed 3.5 × angle 180°	0.41	0.03	0.19	2.77	1.82	0.13
Speed 3.5 × angle 90°	− 0.02	0.03	0.52	− 1.15	1.66	0.49
Speed 3.5 × angle 45°	− 0.02	0.03	0.57	− 1.58	1.73	0.36
Speed 3.5 × angle 0°	1.00	–	–	1.00	–	–
Parameter	Sex					
Girl	− 0.02	0.01	0.01	− 1.48	0.55	0.008
Boy	1.00	–	–	1.00	–	–
Parameter	Covariates					
Stature	0.00	0.00	0.00^a^	0.08	0.04	0.04
Peak $\dot {V}{{\text{O}}_{\text{2}}}$	0.08	0.02	0.00^a^	–	–	–
Scaled $\dot {V}{{\text{O}}_{\text{2}}}$_peak_	–	–	–	− 0.01	0.01	0.32

Reference values speed 2.5 km h^− 1^ and angle set at 0°. ‘×’ Indicates interaction

^a^Significant effect at (*p* < 0.006)

**Table 6:** Straight and turn mean VeDBA models

	Straight mean VeDBA (*g*)			Turn mean VeDBA (*g*)		
	Estimate (*β*)	SE	Sig.	Estimate (*β*)	SE	Sig.
Parameter	Speed					
5.5 km h^−1^	0.35	0.02	0.00^a^	0.38	0.02	0.00^a^
5.5 km h^−1^	0.18	0.02	0.00^a^	0.19	0.01	0.00^a^
5.5 km h^−1^	0.07	0.02	0.00^a^	0.06	0.01	0.00^a^
5.5 km h^−1^	1.00	–	–	1.00	–	–
Parameter			Angle			
180°	0.001	0.01	0.94	0.008	0.01	0.25
90°	− 0.002	0.01	0.86	0.000	0.01	0.98
45°	− 0.001	0.01	0.93	− 0.003	0.01	0.66
0°	1.00	–	–	1.00	–	–
Parameter	Speed × angle					
Speed 5.5 × angle 180°	0.06	0.03	0.04	0.01	0.03	0.60
Speed 5.5 × angle 90°	0.02	0.03	0.50	− 0.01	0.03	0.69
Speed 5.5 × angle 45°	0.04	0.03	0.19	− 0.01	0.03	0.77
Speed 5.5 × angle 0°	1.00	–	–	1.00	–	–
Speed 4.5 × angle 180°	0.04	0.02	0.09	0.03	0.02	0.19
Speed 4.5 × angle 90°	0.01	0.02	0.77	− 0.008	0.02	0.69
Speed 4.5 × angle 45°	0.02	0.02	0.42	− 0.002	0.02	0.92
Speed 4.5 × angle 0°	1.00	–	–	1.00	–	–
Speed 3.5 × angle 180°	0.01	0.02	0.51	0.03	0.01	0.02
Speed 3.5 × angle 90°	0.00	0.02	0.91	0.01	0.01	0.36
Speed 3.5 × angle 45°	0.01	0.02	0.59	0.01	0.01	0.31
Speed 3.5 × angle 0°	1.00	–	–	1.00	–	–
Parameter	Sex					
Girl	0.003	0.01	0.53	0.02	0.00	0.00^a^
Boy	1.00	–	–	1.00	–	–
Parameter	Covariates					
Peak $\dot {V}{{\text{O}}_{\text{2}}}$	− 0.01	0.01	0.11	–	–	–
Scaled $\dot {V}{{\text{O}}_{\text{2}}}$_peak_	–	–	–	0.0002	0.0001	0.10

Reference values speed 2.5 km h^− 1^ and angle set at 0°. ‘×’ Indicates interaction

^a^Significant effect at (*p* < 0.006)

## Abbreviations

BMI: Body mass index
DBA: Dynamic body acceleration
GET: Gas exchange threshold
PHV: Peak height velocity
VeDBA: Vectorial dynamic body acceleration
*C*_r_: The net energy cost of walking
$$\dot {V}{{\text{O}}_{\text{2}}}$$: Oxygen uptake
$\dot {V}{{\text{O}}_{{\text{2}}\,{\text{peak}}}}$: Peak oxygen uptake
6MWT: Six-minute walking test
TCH: Tukey–Ciminera–Heyse
